# Research on influenza epidemic and clinical characteristics based on influenza research database

**DOI:** 10.12669/pjms.40.9.8470

**Published:** 2024-10

**Authors:** Guowei Li, Rongyuan Yang, Rui Chen, Yuejia Zhong, Manhua Huang

**Affiliations:** 1 Guowei Li, Department of Emergency, The Second Affiliated Hospital of Guangzhou University of Chinese Medicine, Guangzhou 510120, Guangdong, China; 2 Rongyuan Yang, Department of Emergency, The Second Affiliated Hospital of Guangzhou University of Chinese Medicine, Guangzhou 510120, Guangdong, China; 3 Rui Chen, Department of Emergency, The Second Affiliated Hospital of Guangzhou University of Chinese Medicine, Guangzhou 510120, Guangdong, China; 4 Yuejia Zhong, Department of Emergency, The Second Affiliated Hospital of Guangzhou University of Chinese Medicine, Guangzhou 510120, Guangdong, China; 5 Manhua Huang, Department of Emergency, The Second Affiliated Hospital of Guangzhou University of Chinese Medicine, Guangzhou 510120, Guangdong, China

**Keywords:** Influenza epidemic, Clinical characteristics, Influenza Research Database, Influenza A virus, Influenza B virus

## Abstract

**Objective:**

To compare the epidemic trends of different types of influenza viruses and the clinical characteristics of patients, so as to provide reference for influenza prevention and control.

**Methods:**

This was descriptive research. The human monitoring data collected from the Influenza Research Database (IRD) from 2006 to 2016 were used to descriptively analyze the distribution of influenza viruses in terms of time, geography, gender and age. The positive samples were divided into three groups based on the type of pathogen (H1N1 influenza A viruses, H3N2 influenza A viruses, and influenza B viruses). Compared and analyzed the distribution and clinical characteristics among groups.

**Results:**

There were statistically significant differences in the positive rates among different countries (*p<* 0.001). The proportion of positive samples gradually decreased with age. The proportion of oseltamivir resistance was significantly higher in H1N1-positive patients compared with that in H3N2-positive patients (*p<* 0.001). Significant differences were observed in the vaccination status among H1N1, H3N2 and influenza B viruses (*p<* 0.001). Cough was common in all cases with H1N1, H3N2 and influenza B infections, while cough, fever and running nose occurred more frequently in influenza B-positive cases than those of H1N1-positive and H3N2-positive cases (*p<* 0.001).

**Conclusion:**

People aged 0-18 years are the major susceptible population to influenza, and H1N1 influenza viruses are the main pathogens of infection in this population, with major clinical manifestations of fever, cough and headache. The findings in this study highlight the necessity to strengthen the protection for this age group in clinical practice.

## INTRODUCTION

Influenza is an acute infectious disease caused by the influenza virus.^1^ Hundreds of millions of influenza virus infections occur every year around the world.^2^ So far, four influenza pandemics have been reported, including two H1N1 pandemics in 1918 and 2009, H2N2 pandemic in 1957 and H3N2 pandemic in 1967. Recent data indicate that a pandemic of influenza will affect 5-15% of the global population, resulting in 4-5 million severe cases and 29,000-65,000 deaths.^3^,^4^ According to the data from the US Centers for Disease Control and Prevention (CDC), the positive rate of influenza viruses was 15.5% during the 2018-2019 flu season, and the weekly detection rate was 1.7%-26.2%.^5^ The main subtype of influenza viruses is H1N1 influenza A virus, followed by H3N2 influenza A virus and influenza B virus. Influenza B viruses, H1N1 and H3N2 influenza A viruses co-circulate in the human population every year, causing seasonal epidemics.^5^

Vaccination against influenza is currently the most effective means of preventing influenza, which can significantly reduce the risk of influenza and severe complications in vaccinators.^6^ However, the evolution of viral antigens has changed the viral proteins previously recognized by the immune system, so that the mutated influenza viruses have an immune escape from the vaccines.^7^ Therefore, the WHO recommends influenza virus vaccine components based on virus strains every season.^8^ The Influenza Research Database(IRD) is commonly used to detect the epidemiological and molecular characteristics of influenza viruses. Its molecular-level data are sourced from public databases such as GenBank, Uniprot, IEDB and PDB. Clinical metadata, monitoring records and serological monitoring records are directly submitted to the IRD through the National Institute of Allergy and Infectious Diseases (NIAID) Project.

As of now, there are few comparative studies on different types of pathogens in influenza patients. Therefore, in the present study, human influenza monitoring data were acquired from the IRD to analyze the epidemiological characteristics of influenza viruses (H1N1 influenza A viruses, H3N2 influenza A viruses, and influenza B viruses) and the clinical symptoms of these patients, so as to provide possible reference for influenza prevention, control, diagnosis and treatment.

## METHODS

This was descriptive research. This study included all cases reported by the IRD from 2006 to 2016 (n=14,735), and positive cases were then screened to establish three groups based on different types of pathogens, i.e., H1N1, H3N2, and influenza B virus. The data used for this study were acquired from the Influenza Research Database (IRD) through http://www.fludb.org,^9^ with duplicates deleted. Samples with missing data were excluded from statistical analysis but included in the analysis of original counting data. The distribution of major influenza viruses in the IRD was investigated in terms of time, geography, gender and age. In addition, this study also collected the clinical characteristics of positive cases to analyze differences in symptoms among different groups.

### Ethical Approval:

The study was approved by the Institutional Ethics Committee of the Second Affiliated Hospital of Guangzhou University of Chinese Medicine (No.: 2020-05-17; date: May 17, 2020), and written informed consent was obtained from all participants.

### Inclusion criteria:

- Patients with influenza-like clinical manifestations, positive throat swabs for influenza A (H1N1) virus nucleic acid test, and imaging findings consistent with influenza manifestations.
- Patients with good communication, informed consent of themselves and their families to the content of this study, and voluntarily signed informed consent.
- Patients with no history of allergy to therapeutic drugs.

### Exclusion criteria:

- Patients with other respiratory diseases, such as asthma.
- Pregnant or lactating women.
- Patients with critical conditions, or with serious primary diseases such as liver and kidney diseases.
- Patients with communication disorders, inability to cooperate with work, and lack of clinical data.
- Patients combined with melancholia.
- Patients with malignant tumors in other parts of the body and patients with metastatic liver cancer.

### Statistical analysis:

The data were statistically analyzed using SPSS 23.0. The qualitative data were expressed as n (%), and analyzed by the c^2^ test. The confidence interval was 95%, paired t-test and independent samples t test were used for comparison between groups. P*<* 0.05 was considered statistically significant.

## RESULTS

**Fig.1:**
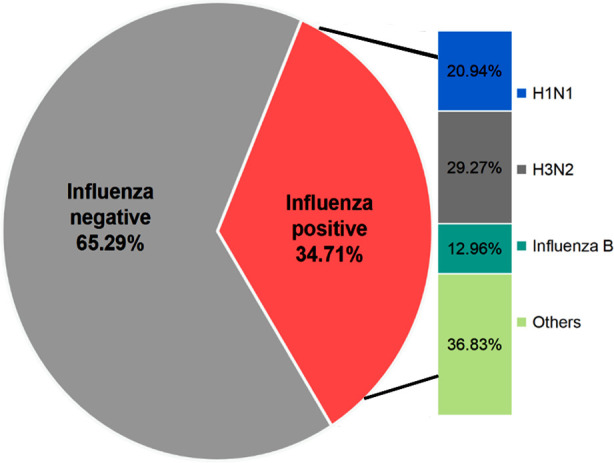
Percentage of influenza types/subtypes reported to Influenza Research Database (IRD) (2006–2016).

**Fig.2:**
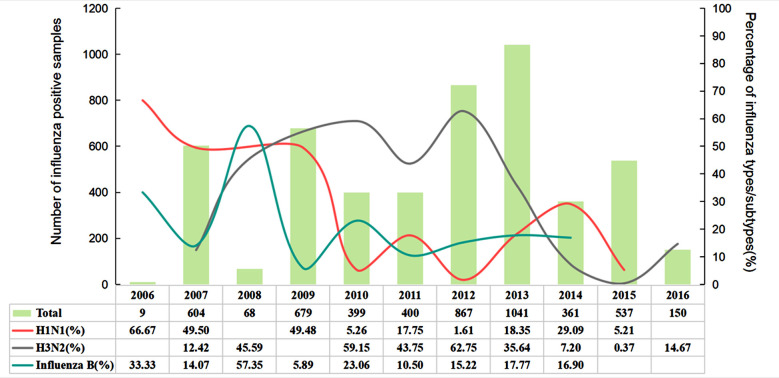
Overall trends in influenza activity reported to IRD for the period 2006 to 2016. The total number of influenza-positive samples received per year is shown as bars. The percentage of positive cases of influenza A H1N1 (pink), H3N2 (gray), and influenza B virus (cyan) reported per year is also presented. Blank cells represent not reported values.

According to Influenza Research Database (IRD) data, a total of 14,735 samples were reported during 2006-2016. Among them, 5,115 cases (about 34.71%) were detected to be influenza virus-positive. The main viruses were H1N1 influenza A viruses, H3N2 influenza A viruses and influenza B viruses, accounting for 20.94%, 29.27% and 12.96% of the total cases, respectively (Fig.1). An annual variation chart was plotted using the data on H1N1, H3N2 and influenza B viruses (Fig.2). During 2006-2016, except that influenza B viruses dominated in 2008, influenza viruses were dominant in all years.

The percentage of positive cases (49.48%) was the highest during the 2009 H1N1 influenza pandemic, with a fluctuating change presented in the following years. H3N2 dominated during 2010-2013 and peaked in 2012. However, in 2014, the percentage of H3N2-positive cases decreased to 7.20%, while the prevalence of H1N1 influenza and influenza B gradually increased, accounting for 29.09% and 16.90% of the positive cases, respectively. After the 2009 pandemic, the percentage of influenza B-positive cases increased rapidly from 5.89% in 2009 to 23.06% in 2010, after which it changed within a constant range of 10%-20%, despite a decline in its change trend. Influenza A viruses were still the main viruses, and H1N1 and H3N2 showed the opposite variation trend (rise in one type but decrease in the other type).

**Fig.3:**
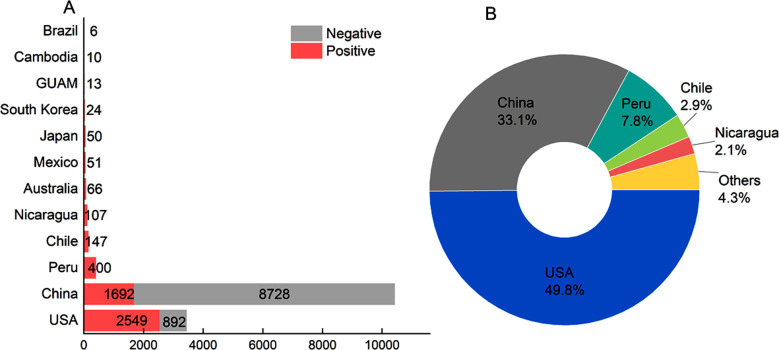
The geographical distribution of influenza viruses.

This study also collected the monitoring records of 12 countries during 2006-2016 from the IRD. In Fig.3A, Fig.3A, the samples from China were the most (70.71%), followed by the USA (23.35%) and Peru (2.71%). Fig.3B displays the percentages of positive samples in different countries, among which 49.83% of the positive cases came from the USA, 33.08% from China, 7.82% from Peru, 2.87% from Chile, and 2.09% from Nicaragua. The proportion of positive cases was 4.3% in other countries (Australia, Mexico, Japan, South Korea, GUAM, Cambodia and Brazil). The differences in the percentage of positive samples were statistically significant among different countries (*p<* 0.001).

Table-I, has the comparison results in the number of detected samples from cases with different ages and genders. The number of detected samples decreased with age, and the proportion of positive cases also decreased. The positive rate was 54.04%, 18.94%, 13.41%, 9.62% and 3.87% in the age groups of 0-18 years, 19-40 years, 41-60 years, 61-80 years and > 80 years, respectively. The proportion of positive samples from females and males was 36.71% and 32.61%, respectively. A statistically significant difference was observed in the detection rate among cases with different ages and genders (*p<* 0.001).

**Table-I:** Distribution of influenza viruses by age and gender [n (%)].

Classification		Positive(n=5,115)	Negative(n=9,620)	c^2^	P
Age (year)	0~18	2,764(54.04)	8,748(90.94)	2775.897	<0.001
	19~40	969(18.94)	480(4.99)		
	41~60	686(13.41)	294(3.06)		
	61~80	492(9.62)	81(0.84)		
	>80	198(3.87)	11(0.11)		
Gender	F	2,492(48.72)	4,297(44.67)	27.070	<0.001
	M	2,561(50.07)	5293(55.02)		

F: female, M: male.

**Fig.4:**
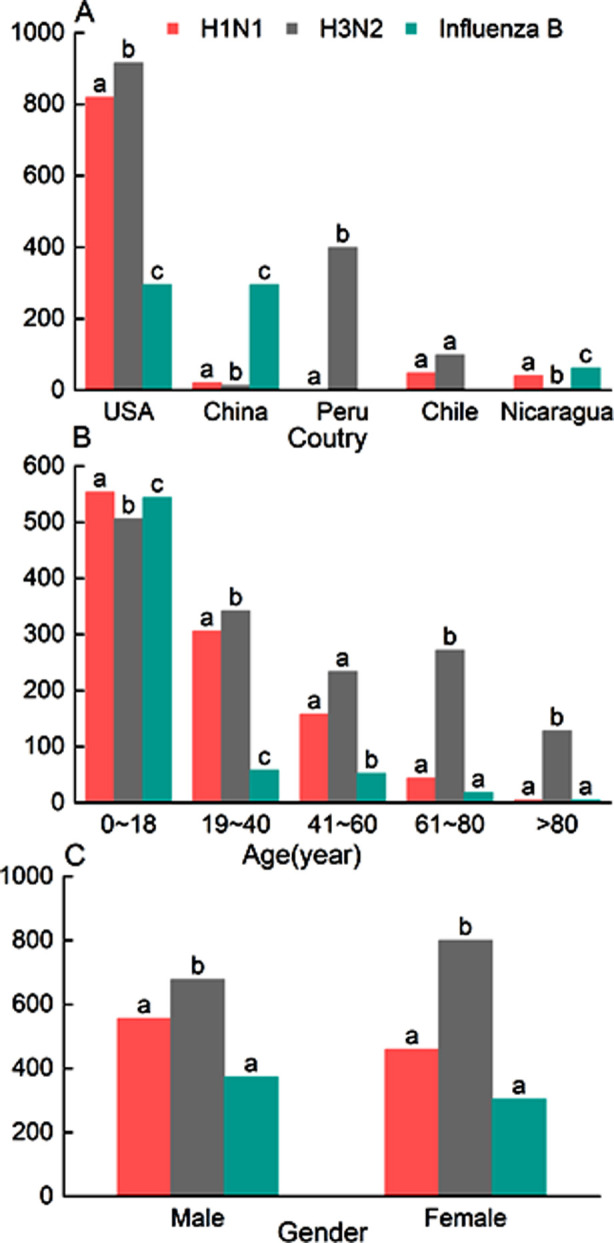
Distribution of country, age and gender in different types of influenza viruses. The same letter indicates no significant differences between groups.

The distribution of H1N1, H3N2 and influenza B viruses reported during 2006-2016 is presented in Fig.4. There were statistically significant differences in the average proportions of H1N1, H3N2 and influenza B viruses among the USA, China, Peru, Chile and Nicaragua (*p<* 0.001; Fig.4A). Except for China and Nicaragua, the number of H3N2-positive cases was dominant in other countries (*p<* 0.05). The percentages of influenza B in China (89.67%) and Nicaragua (57.94%) were significantly higher than those of H1N1 (6.38% and 38.32%, respectively) and H3N2 (3.95% and 3.74%, respectively). Notably, influenza B was not reported in Peru or Chile. Fig.4B exhibits the distribution characteristics of H1N1, H3N2 and influenza B viruses among cases of different ages.

There were significant differences in the prevalence of H1N1, H3N2 and influenza B viruses in people aged 0-18 and 19-40 years (*p<* 0.05), and influenza A (H1N1) viruses were the main pathogen of influenza infection in people aged 0-18 years. The prevalence of H3N2 gradually increased with age, while that of H1N1 and influenza B viruses showed a downward trend. In the populations aged 61-80 years and > 80 years, H3N2 occupied an absolute dominant position (*p<* 0.05), but no significant differences were found in the prevalence of H1N1 and influenza B (*p>* 0.05). The differences in different types of pathogens were also compared between genders (Fig.4C). Among the 5,115 positive cases, there were 1,071 H1N1-positive cases, 1,482 H3N2-positie cases, and 679 influenza B-positive cases. The proportion of H3N2 was the highest in both males and females (42.2% and 46.6%, respectively), which was also significantly higher than that of H1N1 and influenza B. However, no statistically significant difference was detected in the prevalence of H1N1 and influenza B between males and females (*p>* 0.05).

There were statistically significant differences in oseltamivir resistance and vaccination among various types of influenza viruses (*p<* 0.001; Table-II). The proportion of oseltamivir resistance was significantly higher in H1N1-positive patients compared with that of H3N2-positive patients (*p<* 0.001). The pairwise comparison also revealed significant differences in vaccination status among H1N1, H3N2 and influenza B viruses (*p<* 0.001). Among them, the percentage of H3N2-positive, H1N1-positive and B influenza-positive patients with vaccination history was 21.79%, 19.89% and 16.94%, respectively. While no significant difference was noticed in the proportion of people with onset hours ≥ 24 among the three types/subtypes (*p>* 0.05).

**Table-II:** Oseltamivir resistance, vaccination status and onset hour of different types of influenza viruses [n (%)]

Items	H1N1 (n=1,071)	H3N2 (n=1,482)	Influenza B (n=679)	c^2^	P
Oseltamivir resistance	80 (7.47)	1 (0.07)	/	136.722	<0.001
Get vaccinated	213 (19.89) a	323 (21.79) b	115 (16.94) c	67.68	<0.001
Onset Hours≥24h	369 (34.45)	108 (7.29)	71 (10.46)	1.375	0.503

Table-III summarizes the clinical symptoms of cases with H1N1, H3N2, and influenza B infections. The most common clinical symptoms of H1N1-positive patients were cough (52.38%) and fever (47.15%), followed by headache (36.32%), vomiting (33.15%), fatigue (31.56%) and running nose (30.25%). The proportions of cough (57.83%), running nose (38.66%), headache (31.11%), throat (29.76%) and fever (27.6%) were relatively high in H3N2-infected patients. Meanwhile, the most common clinical symptoms of people infected with influenza B viruses were fever (82.47%), cough (52.38%) and running nose (64.21%), followed by throat (45.51%) and headache (30.34%). Except for chest pain, earache and rash, significant differences were detected in other clinical symptoms among patients infected with different types of pathogens (*p<* 0.001).

**Table-III:** Distribution of clinical symptoms in different types of influenza viruses [n (%)].

Symptoms	H1N1 (n=1,071)	H3N2 (n=1,482)	Influenza B (n=679)	c^2^	P
Aches	231(21.57)	152(10.26)	94(13.84)	83.666	<0.001
Chest pain	74(6.91)	49(3.31)	39(5.74)	0.910	0.634
Chills	227(21.20)	194(13.09)	160(23.56)	6.241	0.044
Conjunctivitis	53(4.95)	86(5.80)	19(2.80)	35.085	<0.001
Cough	561(52.38)	857(57.83)	517(76.14)	11.545	0.003
Diarrhea	78(7.28)	28(1.89)	52(7.66)	6.352	0.042
Earache	50(4.67)	31(2.09)	35(5.15)	0.706	0.702
Fatigue	338(31.56)	200(13.50)	143(21.06)	34.103	<0.001
Fever	505(47.15)	409(27.60)	560(82.47)	133.415	<0.001
Headache	389(36.32)	461(31.11)	206(30.34)	34.245	<0.001
Malaise	10(0.93)	3(0.20)	51(7.51)	137.980	<0.001
Myalgia	148(13.82)	253(17.07)	86(12.67)	9.975	0.007
Nausea	114(10.64)	69(4.66)	76(11.19)	10.100	0.006
Rash	8(0.75)	3(0.20)	2(0.29)	1.149	0.563
Running nose	324(30.25)	573(38.66)	436(64.21)	42.671	<0.001
Short breath	5(0.47)	5(0.34)	33(4.86)	44.053	<0.001
Sinus congestion	185(17.27)	142(9.58)	134(19.73)	9.283	0.010
Throat	32(2.99)	441(29.76)	309(45.51)	204.364	<0.001
Vomiting	355(33.15)	37(2.50)	36(5.30)	177.930	<0.001
Wheezing	60(5.60)	2(0.13)	37(5.45)	45.431	<0.001

## DISCUSSION

During 2006-2016, a total of 14,735 samples were collected, with a positive rate of 34.71% (n=5,115), which is far higher than 14.7% reported in South Korea during 2009-2016^10^ and 22.2% in Madrid during 2010-2016^11^, but lower than 36% in Mexico during 2010-2016.^12^ The relatively higher positive rate of this study may be explained by the limited sample source, for example, the IRD only includes Australia, Brazil, Cambodia, GUAM, Japan, Mexico, South Korea, Zambia, the USA, China, Peru, Chile, Nicaragua, etc. Among the 5,115 positive samples, over 50% of the positive cases (50.21%) were influenza A virus-positive, of which H1N1 and H3N2 accounted for higher proportions (20.94% and 29.27%, respectively), and influenza B viruses accounted for 12.96%. Different types of viruses differed in infectivity and transmissibility, but the overall epidemic activity of influenza viruses was higher than that of influenza B viruses. Additionally, H1N1 and H3N2 exhibited synchronization of opposite trend, i.e., low H1N1 and high H3N2.

Similarly, Chiu et al.^13^ analyzed the variation characteristics of influenza A (H1N1) and influenza A (H3N2) viruses in Hong Kong, with the discovery of an antiphase synchronization as well. The transmission dynamics of influenza viruses are mainly caused by the changes in viral antigens and are also influenced by climate, regional characteristics, human factors, etc.^14^ According to IRD data, China and the USA had the highest number of detected cases (9,970 and 3,441, respectively), with positive rates of 33.1% and 49.8%, respectively. A study reported that the positive rate of influenza A viruses in Macau, China during 2010-2018 was 17.17%, while that of influenza B viruses was 6.97%.^15^ Compared with other years, the positive rate was roughly parallel to that of the USA (52.2%) during 2018-2019.^5^

It has been reported that during the COVID-19 pandemic, the incidence of influenza declined significantly, and the epidemic strain also changed from Type-A to Type-B^16^, which may be related to the epidemic prevention and control policy, population immunity and viral evolution.^13^ In our study, minors (0-18 years old) accounted for 54.04% of the positive cases, significantly higher than those aged 19-40 years (18.94%), 41-60 years (13.41%), 61-80 years (9.62%) and >80 years (3.87%). H1N1 and influenza B infections were more prevalent among minors, while H3N2 infection was more common in other age groups. In general, influenza B viruses have a slower variation rate and are more common in children,^1^,^17^ which can be attributed to frequent interpersonal contact, hygiene habits, and hypoimmunity.^18^ In addition, there were statistically significant differences in oseltamivir resistance and vaccination among cases with different influenza types/subtypes (H1N1, H3N2 and influenza B). Specifically, 7.47% of H1N1-positive patients and 0.07% of H3N2-positive patients were resistant to oseltamivir, respectively. Drug resistance monitoring in influenza viruses may benefit a reasonable control of the prescription of antiviral drugs by clinicians.^19^

Moreover, the average vaccination rate was 19.54% among all the positive samples, with the highest rate detected in H3N2-positive cases, followed by H1N1-positive cases. Clinically, influenza vaccination has been accepted to be the optimal intervention to reduce hospitalization rates and mortality. Nevertheless, the vaccination rate may be influenced by many factors, such as the local medical system, income level, and awareness of influenza and immunity to influenza. The vaccination rate against influenza in developed countries ranges from 30% to 60%^20^, which, however, is only 4% in developing countries.^21^ Cough was common in all cases with H1N1, H3N2 and influenza B infections, and subjects with influenza B infection may frequently occur cough, fever and running nose compared to those with H1N1 and H3N2 infections. In addition, some researchers noticed that the main symptoms of influenza infection included fever, cough, muscle and joint pain,^18^ fever, sore throat, muscle pain and weakness.^22^ Moreover, symptoms of influenza vary among individuals, which may be related to different ages, countries or regions, and types of pathogen.^23^

### Limitations:

The monitoring in the IRD was conducted on an annual basis, which was impossible to summarize the seasonal patterns of influenza. Additionally, despite the inclusion of comprehensive clinical symptoms in the IRD, there might still be biased analysis results due to the differences in testing policies.

## CONCLUSION

Influenza A viruses are dominant pathogens, and there is anti-phase synchronization between H1N1 and H3N2. The prevalence of H1N1 is higher in people aged 0-18 years, while that of H3N2 is higher in people aged 19-40 years and >60 years. Collectively, findings in this study suggest targeted prevention and control for potential influenza epidemics in populations of different ages based on the epidemic activity of relevant viruses.

### Authors’ Contributions:

**GL and RY:** Carried out the studies, participated in collecting data, and drafted the manuscript, and are responsible and accountable for the accuracy or integrity of the work.

**RC:** Performed the statistical analysis and participated in its design.

**YZ and MH:** Participated in acquisition, analysis, or interpretation of data and draft the manuscript.

All authors read and approved the final manuscript.
